# The risk of deliberate self-harm following a diagnosis of rheumatoid arthritis or ankylosing spondylitis: A population-based cohort study

**DOI:** 10.1371/journal.pone.0229273

**Published:** 2020-02-21

**Authors:** Bindee Kuriya, Simone Vigod, Jin Luo, Jessica Widdifield, Nigil Haroon

**Affiliations:** 1 Sinai Health System, University of Toronto, Toronto, Ontario, Canada; 2 Women’s College Hospital, University of Toronto, Toronto, Ontario, Canada; 3 ICES, Toronto, Ontario, Canada; 4 Holland Musculoskeletal Research Program, Sunnybrook Research Institute, Toronto, Ontario, Canada; 5 Institute of Health Policy, Management & Evaluation, University of Toronto, Toronto, Ontario, Canada; 6 Krembil Research Institute, University Health Network, University of Toronto, Toronto, Ontario, Canada; Universita Campus Bio-Medico di Roma, ITALY

## Abstract

**Objective:**

Rheumatoid arthritis (RA) and ankylosing spondylitis (AS) are associated with mental illness. The risk of serious mental illness, including deliberate self-harm (DSH), in these conditions is not well known. We aimed to determine if RA or AS independently increases the risk for DSH.

**Methods:**

We conducted retrospective, population-based cohort studies using administrative health data for the province of Ontario, Canada between April 1, 2002 and March 31, 2014. Individuals with incident RA (N = 53,240) or AS (N = 13,964) were separately matched 1:4 by age, sex, and year with comparators without RA or AS. The outcome was a first DSH attempt identified using emergency department data. We estimated hazard ratios (HR) and 95% confidence intervals (95% CI) for risk of DSH in RA and AS versus comparators, adjusting for demographic, clinical and health service utilization variables.

**Results:**

Subjects with AS were significantly more likely to self-harm (crude incidence rate [IR] of 0.68/1,000 person years [PY] versus 0.32/1,000 PY in comparators), with an adjusted HR of 1.59 (95% CI 1.15 to 2.21). DSH was increased for RA subjects (IR 0.35/1,000 PY) versus comparators (IR 0.24/1,000 PY) only before (HR 1.43, 95% CI 1.16 to 1.74), but not after covariate adjustment (HR 1.07, 95% CI 0.86 to 1.33).

**Conclusions:**

AS carries an increased risk for DSH but no such risk was observed in RA. Further evaluation of at-risk AS subjects is needed, including the longitudinal effects of disease and arthritis therapies on self-harm behaviour. This will inform whether specific risk-reduction strategies for DSH in inflammatory arthritis are needed.

## Introduction

Individuals with inflammatory arthritis (IA) experience significant psychological burden related to their illness [1, 2]. Systemic inflammation and immune dysregulation, central to the pathogenesis of IA, has been postulated to have direct effects on neural signaling and mood [3–5]. Moreover, the physical effects of pain, disability and fatigue may contribute to feelings of depression, anxiety, hopelessness and social isolation [2, 6, 7].

Rheumatoid arthritis (RA) is the most common IA affecting peripheral joints, with worldwide prevalence of 1% [8]. Ankylosing spondylitis (AS) is far less prevalent (0.3%), with cardinal features of inflammatory back pain and stiffness, primarily in young men [9]. Despite their different clinical phenotypes, RA and AS are both associated with an elevated risk of mental illness, including depression, anxiety and substance abuse [2, 10–13]. An increased risk of completed suicide has also been described for these groups and may relate to struggles with arthritis symptoms, mental illness or a combination of both [11, 13].

Deliberate self-harm (DSH), is intentional self-injury or self-poisoning that increases the risk of death by suicide [14–16]. DSH is important to recognize because repeated DSH attempts are common and frequently more severe than the initial attempt [17–19]. The risk of DSH is elevated in other chronic conditions such as diabetes and multiple sclerosis [19]. However, the associations between RA or AS and the risk of DSH are not well known, especially following disease onset, and in the absence of diagnosed mental illness. If a link between these inflammatory conditions and DSH exists, efforts to screen and triage these populations for timely specialty care at the time of diagnosis would be justified [20].

We tested our hypothesis that IA diagnosis increases the risk of DSH. Thus, the aims of this study were to examine = two distinct IA conditions to: (1) estimate the incidence of a first DSH attempt following RA or AS diagnosis, and (2) determine if RA or AS are independently associated with DSH, even in the absence of a previous history of mental illness, compared to the general population.

## Patients and methods

### Study design and study setting

We conducted retrospective, cohort studies using health administrative data for the province of Ontario, Canada. Data were obtained from ICES (www.ices.on.ca), using five databases: (1) The Registered Persons Database contains information on date of birth, sex, postal code and vital status; (2) The Ontario Health Insurance Plan Claims History Database identifies diagnoses and procedures associated with physician billing claims; (3) The Canadian Institutes of Health Information Discharge Abstract Database compiles information for all hospital admissions and medical discharge records are coded using diagnosis codes (ICD-9, the International Classification of Diseases−9th revision and ICD-10, the Canadian version of the 10th revision); (4) The Ontario Mental Health Reporting System contains clinical and administrative data on all mental health hospitalizations and; (5) the National Ambulatory Care Reporting System identifies all emergency department (ED) visits and day surgeries. These datasets were de-identified and linked using unique encoded identifiers and analyzed at the ICES. The use of data in this project was authorized under section 45 of Ontario’s Personal Health Information Protection Act, which does not require review by a Research Ethics Board.

### Participants

Two parallel matched cohorts were analyzed. In the first, we compared individuals with incident RA to a matched, non-RA comparator cohort. In the second, we compared individuals with incident AS to a separate matched non-AS comparator cohort.

All subjects (RA, AS, and controls) were excluded if they had any mental illness or addiction encounters with a family physician (by outpatient billing codes ICD-9: 290–315) or psychiatrist, identified through the provider specialty code, in the previous two years [21]. Subjects were further excluded for any hospitalization or ED visit for a mental health diagnosis, including DSH, (ICD-10:F04-F99, ICD-10: X60-X84, Y10-Y19, Y28) in the two years prior to index date.

We identified an inception RA cohort within the validated Ontario Rheumatoid Arthritis Dataset (ORAD) from April 1, 2002 to March 31, 2014 that has been described in detail [22, 23]. Patients were excluded if they had missing age or sex, or other connective tissue diseases (systemic lupus erythematosus systemic sclerosis, Sjogren’s syndrome, inflammatory myositis; ICD-9 710, ICD-10 M32-M36), psoriatic arthritis (ICD-9:696, ICD-10:L40) or ankylosing spondylitis (ICD-9:720) in the two years before index date. The comparison cohort comprised Ontarians without RA, matched 4:1 on age (±365 days), sex and calendar year. Comparators were randomly assigned an index date matching the distribution of the corresponding RA cases by calendar year and quarter.

For the second matched cohort, we used a published algorithm to identify an incident AS cohort [9]. The diagnosis of AS was based on billing data and could not distinguish between radiographic and non-radiographic AS. Patients were excluded if missing age or sex, other connective tissue diseases (systemic lupus erythematosus, systemic sclerosis, Sjogren’s syndrome, and myositis), psoriatic arthritis or RA in the two years before index date. The comparison cohort included Ontarians without AS, matched 4:1 to AS patients on age (±365 days), sex and calendar year. Index date for comparators was randomly assigned to match the distribution of AS cases by calendar year and quarter.

All subjects were followed from cohort entry date until migration, death, end of study period (31 March 2016), or outcome occurrence, whichever came first.

### Outcome

Our outcome of interest was a first ED presentation for DSH attempt with disposition alive or dead. A priori, we opted not to look at completed suicide on its own due to expected small numbers leading to an underpowered estimate. We used a validated definition of DSH: any ICD-10 diagnostic code for intentional self-harm (ICD-10: X60-84), or any code for self-poisoning, (ICD-10:Y10-19) or contact with sharp object (ICD-10:Y28) [14, 15]. Information on disposition from the ED was characterized as admitted to hospital, transferred to another ED, left without treatment/against medical advice, discharged from the ED or death. Disposition events with an absolute number fewer than six in number, including deaths, were not reported due to ICES privacy regulations.

### Covariates

In addition to age and sex, we collected place of residence (urban versus rural), median household income and ethnicity (Chinese, South Asian and Other [primarily Caucasian]) with a validated surname algorithm [24]. Baseline comorbidities were collected using a 24-month look back window. We used validated algorithms to identify hypertension [25], diabetes [26], inflammatory bowel disease [27], coronary artery disease and acute myocardial infarction [28], cerebrovascular accident [29], chronic obstructive pulmonary disease /asthma [30], renal failure [31], cancer, upper gastrointestinal bleed, osteoarthritis, osteoporosis, infection, or psoriasis [9]. The Charlson Comorbidity Index was used as composite measure of illness burden [32, 33]. Non-mental health care utilization in the two years before index date was also examined according to the number of outpatient visits, rheumatology visits, hospitalizations and ED visits.

### Statistical analysis

We used standardized differences to compare baseline characteristics of individuals with RA, AS and matched comparators [34].

We constructed cumulative incidence curves, stratified by sex, and tested for differences in incidence rates using the log-rank test. We calculated crude incidence rates and 95% confidence intervals (95% CI) for DSH, per 1,000 person-years, for RA, AS and matched comparators.

Cox proportional hazards models with Bonferroni correction were used to estimate hazards ratios (HR) and 95% CI for RA and AS, separately. Clinically important confounders of the association between RA or AS and self-harm were a priori selected to be age and sex. Therefore, we matched our populations by age and sex so these variables were not then entered into statistical models. First we generated unadjusted hazard ratios for the association between RA or AS and DSH. Then, we constructed univariable models including all baseline variables. The full multivariable models adjusted for income, ethnicity, and previous physician and ED visits (deemed to be clinically important confounders). In addition, variables with p<0.05 in univariable models were included: a history of psoriasis, or a history of chronic obstructive pulmonary disease in the RA model; and a history of infection in the AS model. All analyses were conducted using SAS, version 9.3 (SAS Institute).

## Results

**Fig 1:**
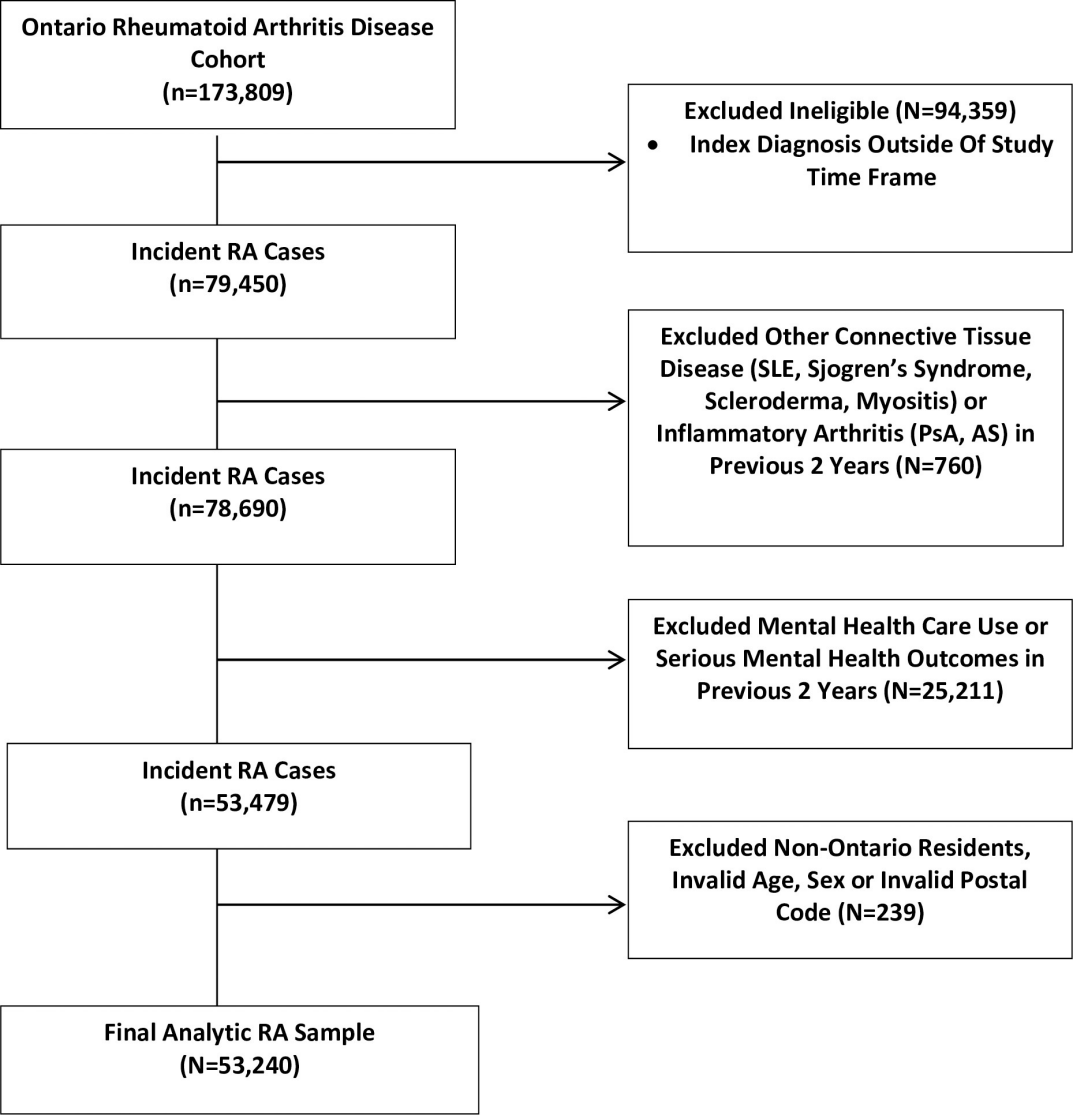
Flow chart of RA cohort creation. AS, ankylosing spondylitis; PsA, psoriatic arthritis; RA, rheumatoid arthritis; SLE, systemic lupus erythematosus.

**Fig 2:**
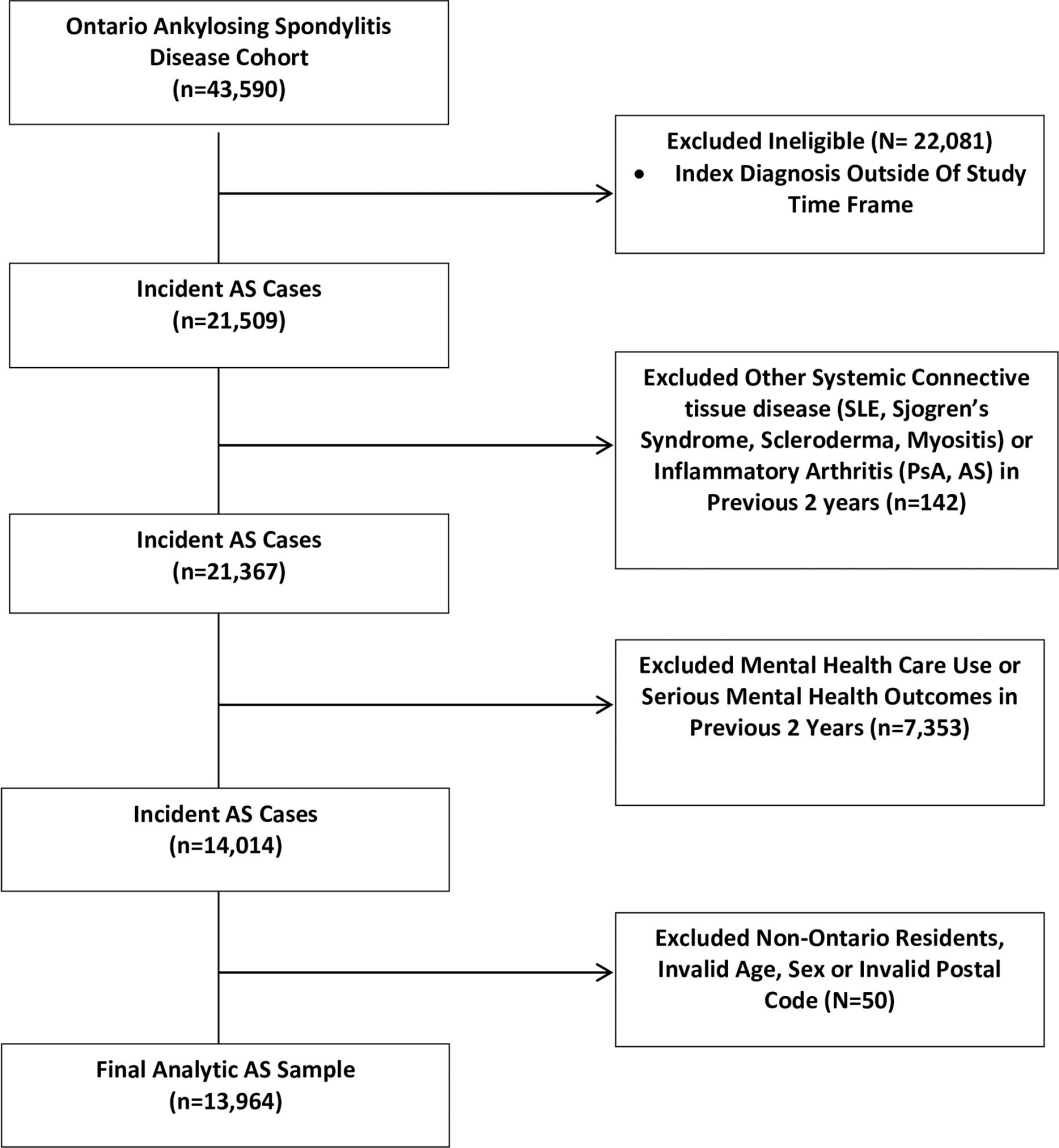
Flow chart of AS cohort creation. AS, ankylosing spondylitis; PsA, psoriatic arthritis; RA, rheumatoid arthritis; SLE, systemic lupus erythematosus.

A total of 53,240 Ontarians with RA (matched to 212,960 comparators) and 13,964 individuals with AS (matched to 55,856 comparators) were included in the analyses (Figs 1 and 2). Table 1 depicts baseline characteristics of the cohorts. Mean age of RA patients was 56.6 years (SD, 17.1) and 67% were female. Patients with AS were younger, with a mean age of 46.4 years (SD, 16.6) and 43% were female. Patients with RA or AS were not significantly different than their matched comparators with respect to demographic variables but did have a greater number of comorbid conditions. Subjects with RA and AS also had greater non-mental health care use (Table 1).

**Table 1:** Baseline characteristics of individuals with RA, AS or matched comparators who attended the emergency department for a deliberate self-harm attempt.

Variable	RA n = 53,240	Non-RA Comparator n = 212,960	Standardized Difference	AS n = 13,964	Non-AS Comparator n = 55,856	Standardized Difference
**Demographics**						
Age at index date, mean ± SD	56.5 ± 17.1	56.5 ± 17.1	0	46.4 ± 16.6	46.4 ± 16.6	0
Female, n (%)	35,702 (67.1)	142,808 (67.1)	0	6,064 (43.4)	24,256 (43.4)	0
Rural residence, n (%)	7,743 (14.5)	25,771 (12.1)	0.07	1,608 (11.5)	6,216 (11.1)	0.01
Income quintile, n (%)						
1 (lowest)	9,878 (18.6)	41,582 (19.5)	0.02	2,359 (16.9)	11,275 (20.2)	0.08
2	10,777 (20.2)	42,800 (20.1)	0	2,595 (18.6)	11,237 (20.1)	0.04
3	10,930 (20.5)	41,616 (19.5)	0.02	2,715 (19.4)	11,038 (19.8)	0.01
4	10,907 (20.5)	42,697 (20.0)	0.01	3,056 (21.9)	11.163 (20.0)	0.05
5	10,748 (20.2)	44,265 (20.8)	0.01	3,239 (23.2)	11.143 (19.9)	0.08
Ethnicity, n (%)						
Chinese	1,279 (2.4)	12,943 (6.1)	0.18	709 (5.1)	3.677 (6.6)	0.06
South Asian	1,980 (3.7)	6,820 (3.2)	0.03	434 (3.1)	2,070 (3.7)	0.03
Other	49,981 (93.9)	193,197 (90.7)	0.12	12,821 (91.8)	50,109 (89.7)	0.07
**Comorbidity, n (%)**						
Charlson Score						
0	48,895 (91.8)	202,214 (95.0)	0.13	13,211 (94.6)	54,237 (97.1)	0.13
1	2,131 (4.0)	4,569 (2.1)	0.11	426 (3.1)	725 (1.3)	0.12
2	1,260 (2.4)	3,440 (1.6)	0.05	207 (1.5)	517 (0.9)	0.05
3+	954 (1.8)	2,737 (1.3)	0.04	120 (0.9)	377 (0.7)	0.02
OA	15,200 (28.5)	11,602 (5.4)	0.65	2,164 (15.5)	1,658 (3.0)	0.44
OP	1,153 (2.2)	3,286 (1.5)	0.05	237 (1.7)	370 (0.7)	0.10
COPD	6,970 (13.1)	18,334 (8.6)	0.14	984 (7.0)	2,791 (5.0)	0.09
CAD	2,807 (5.3)	7,912 (3.7)	0.08	434 (3.1)	1,190 (2.1)	0.06
MI	502 (0.9)	1,351 (0.6)	0.03	68 (0.5)	203 (0.4)	0.02
CVD	114 (0.2)	447 (0.2)	0	17 (0.1)	51 (0.1)	0.01
HTN	21,674 (40.7)	70,533 (33.1)	0.16	3,663 (26.2)	11,082 (19.8)	0.15
ARF	594 (1.1)	1,170 (0.5)	0.06	107 (0.8)	195 (0.3)	0.06
CRF	1,284 (2.4)	2,757 (1.3)	0.08	224 (1.6)	433 (0.8)	0.08
Diabetes	8,161 (15.3)	27,304 (12.8)	0.07	1,473 (10.5)	4,622 (8.3)	0.08
Dementia	177 (0.3)	645 (0.3)	0.01	31 (0.2)	59 (0.1)	0.03
Malignancy	3,539 (6.6)	13,088 (6.1)	0.02	590 (4.2)	1,827 (3.3)	0.05
UGIB	142 (0.3)	249 (0.1)	0.03	17 (0.1)	43 (0.1)	0.01
Infection	23,051 (43.3)	58,943 (27.7)	0.33	6,049 (43.3)	14,197 (25.4)	0.38
IBD	827 (1.6)	1,064 (0.5)	0.10	778 (5.6)	230 (0.4)	0.31
Psoriasis	643 (1.2)	393 (0.2)	0.12	399 (2.9)	81 (0.1)	0.22
**Non-Mental Health Care Utilization**						
Physician visits, n (%)	52,290 (98.2)	163,790 (76.9)	0.68	13,830 (99.0)	39,476 (70.7)	0.86
Physician visits, mean ± SD	29.2 ± 26.92	15.3 ± 20.9	0.57	26.7 ± 25.4	11.01 ± 18.02	0.71
Rheumatology visits, n (%)	18,196 (34.2)	8,602 (4.0)	0.83	4,967 (35.6)	1,537 (2.8)	0.92
Rheumatology visits, mean ± SD	1.31 ± 3.32	0.09 ± 0.67	0.51	1.37 ± 3.77	0.08 ± 0.86	0.47
Hospitalizations, n (%)	8,388 (15.8)	18,862 (8.9)	0.21	1,735 (12.4)	3,551 (6.4)	0.21
Hospitalizations, mean ± SD	0.24 ± 0.70	0.13 ± 0.51	0.18	0.18 ± 0.62	0.09 ± 0.41	0.18
ED visit, n (%)	23,586 (44.3)	49,702 (23.3)	0.45	6,068 (43.5)	12,432 (22.3)	0.46
ED visit, mean ± SD	1.01 ± 1.88	0.44 ± 1.23	0.36	0.99 ± 2.07	0.41 ± 1.16	0.35

ARF, acute renal failure; AS, ankylosing spondylitis; CAD, coronary artery disease; COPD, chronic obstructive pulmonary disease; CRF, chronic renal failure; CVD, cardiovascular disease; ED, emergency department; IBD, inflammatory bowel disease; MI, myocardial infarction, OA, osteoarthritis; OP, osteoporosis; RA, rheumatoid arthritis, SD, standard deviation, UGIB, upper gastrointestinal bleed

The RA cohort generated 367,468 person-years (PY) of follow-up and 129 subjects with RA had a DSH attempt compared to 372 in the comparator group (1,520,397 PY). There were 69 DSH attempts in the AS group (101,633 PY) compared to 131 in the non-AS cohort (411,198 PY) (Table 2).

**Table 2:** Crude incidence rates of deliberate self-harm attempt and disposition following emergency department presentations in RA, AS and matched comparators.

	Number of Events	Person-Years at Risk	Crude Incidence Rate Per 1,000 PY (95% CI)	Admitted, n (%)	Discharged, n (%)	Transferred or Left Early, n (%)
**RA**	129	367,468	0.35 (0.29–0.42)	29 (22%)	89 (69%)	6 (5%)
**Non-RA Comparator**	372	1,520,397	0.24 (0.22–0.27)	49 (13%)	270 (73%)	51 (14%)
**AS**	69	101,633	0.68 (0.53–0.86)	10 (15%)	53 (77%)	6 (8%)
**Non-AS Comparator**	131	411,198	0.32 (0.27–0.38)	20 (16%)	97 (75%)	8 (6%)

AS, ankylosing spondylitis; RA, rheumatoid arthritis; PY, person-year; 95% CI, 95% confidence intervals. Disposition events with absolute number <6, including death, were not reported due to privacy regulations, therefore percentages may not equal 100%.

**Fig 3:**
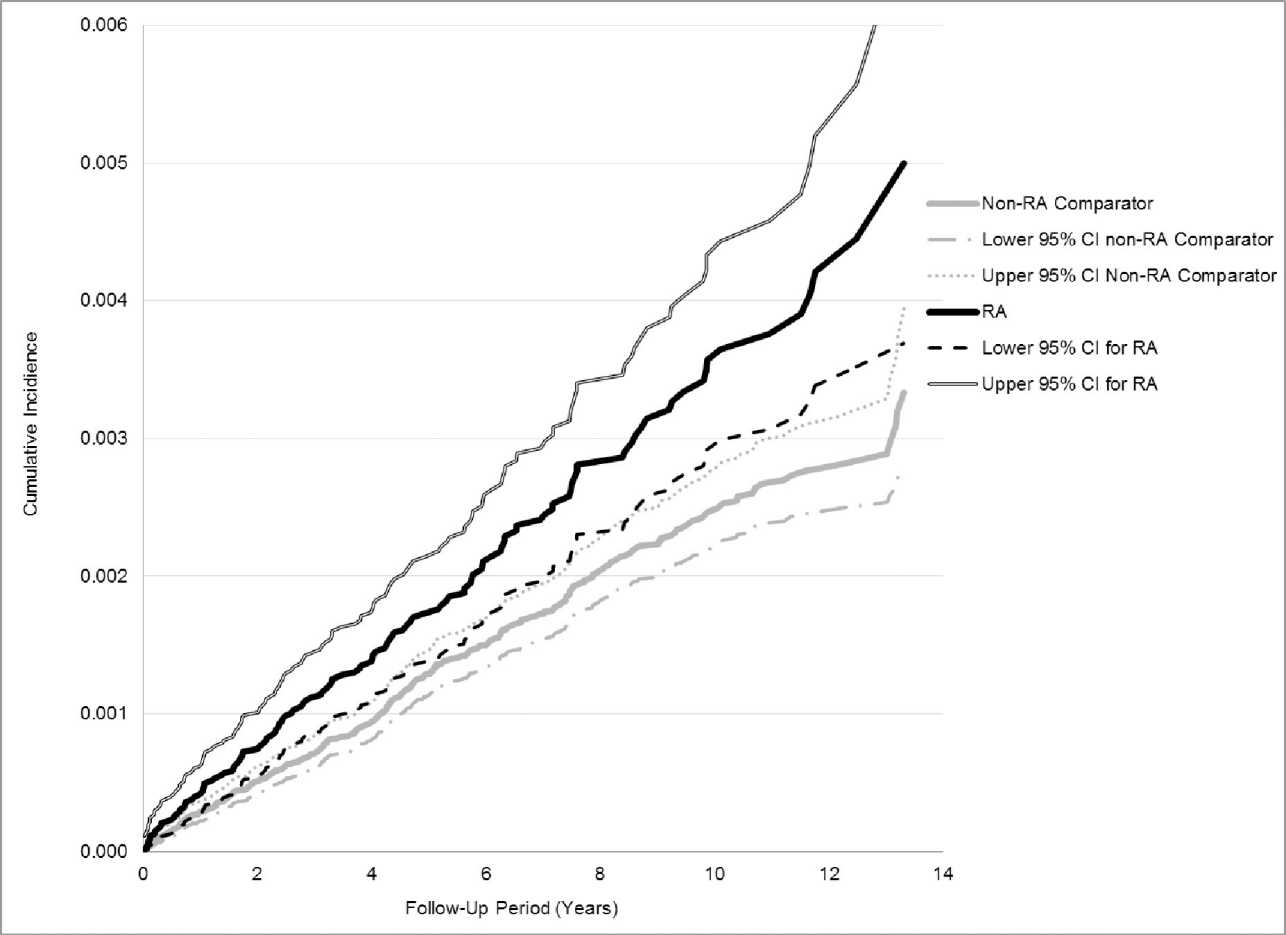
Cumulative incidence of a first deliberate self-harm attempt, over the study period years 2002 to 2014, in subjects with RA and matched comparators.

**Fig 4:**
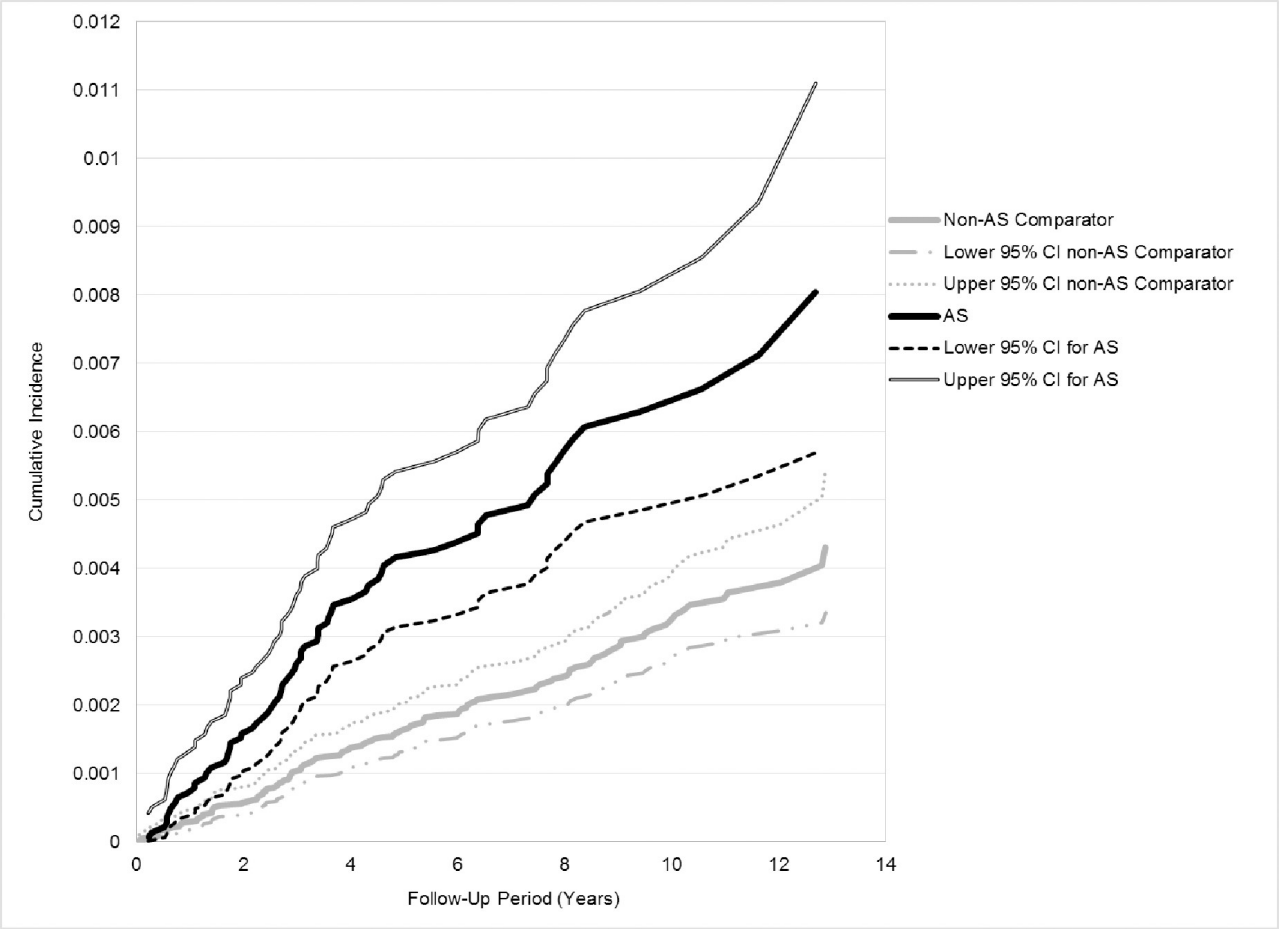
Cumulative incidence of a first deliberate self-harm attempt, over the study period years 2002 to 2014, in subjects with AS and matched comparators.

RA patients had a higher cumulative crude incidence of DSH compared to non-RA subjects (p <0.0004, Fig 3). The crude incidence rate (IR) of DSH among RA subjects was 0.35/1,000 PY versus the comparator group rate of 0.24/1,000 PY (Table 2). Individuals with AS had a greater cumulative incidence of DSH (p<0.0001, Fig 4) and a higher DSH rate (IR 0.68/1,000 PY) than comparators (IR of 0.32/1,000 PY, Table 2).

The most frequent method of self-harm was self-poisoning in both the AS and RA groups. Poisoning occurred in 64% of AS self-harm attempts versus 66% in comparators, and in 81% of attempts in RA versus 65% in comparators. The second most common method was contact with a sharp object/self-cutting, which occurred in 32% of attempts in AS versus 29% in comparators, and 16% of attempts in RA versus 31% in comparators. Following presentation, the majority of subjects (range 69% to 77%) were discharged directly from the ED (Table 2).

In analyses adjusted for baseline demographics, comorbidity and health care utilization, the hazard for DSH was attenuated in the AS cohort (Table 3) but remained statistically significant (HR 1.59, 95% CI 1.15 to 2.21) after full covariate adjustment. The increased risk of DSH for RA subjects was attenuated after adjusting for baseline factors.

**Table 3:** Unadjusted, univariable and multivariable adjusted hazard ratios (HR) with 95% confidence intervals (95% CI) for a deliberate self-harm attempt among individuals with rheumatoid arthritis or ankylosing spondylitis.

Variable	Rheumatoid Arthritis			Ankylosing Spondylitis		
	Unadjusted HR (95% CI)	Univariable Adjusted HR^a^ (95% CI)	Multivariable Adjusted HR^b^ (95% CI)	Unadjusted HR (95% CI)	Univariable Adjusted HR^a^ (95% CI)	Multivariable Adjusted HR^b^ (95% CI)
RA	***1*.*43 (1*.*16–1*.*74)***	1.07 (0.82–1.38)	1.07(0.86–1.33)			
AS				***2*.*09 (1*.*56–2*.*81)***	***1*.*78 (1*.*21–2*.*63)***	***1*.*59 (1*.*15–2*.*21)***
**Demographics**						
Rural residence		1.28 (0.95–1.72)			0.65 (0.34–1.23)	
Income quintile						
2		0.80 (0.58–1.09)	0.80 (0.59–1.08)		0.39 (0.28–0.68)	0.40 (0.24–0.68)
3		0.82 (0.61–1.12)	0.82 (0.61–1.11)		0.74 (0.44–1.23)	0.79 (0.49–1.29)
4		0.69 (0.50–0.95)	0.70 (0.51–0.96)		0.58 (0.35–0.99)	0.55 (0.33–0.91)
5		0.53 (0.38–0.74)	0.56 (0.40–0.77)		0.50 (0.29–0.87)	0.49 (0.29–0.83)
Ethnicity						
Chinese		0.58 (0.31–1.06)	0.56 (0.31–1.03)		0.27 (0.08–0.91)	0.35 (0.12–1.03)
South Asian		1.56 (0.92–2.65)	1.47 (0.87–2.49)		0.16 (0.03–0.73)	0.17 (0.04–0.77)
**Comorbidity**						
Charlson Score						
1		1.60 (0.72–3.54)			0.78 (0.13–4.71)	
2		0.50 (0.17–1.50)			0	
3+		3.89 (0.67–22.42)			0	
OA		0.92 (0.62–1.38)			0.91 (0.34–2.43)	
OP		0.22 (0.05–0.99)	0.25 (0.05–1.11)		0	
COPD		1.54 (1.01–2.35)	1.75 (1.18–2.01)		2.05 (0.68–6.21)	
CAD		0.48 (0.20–1.12)			0	
MI		0.89 (0.13–6.30)			0	
Hypertension		0.79 (0.58–1.09			1.36 (0.70–2.63)	
ARF		0.86 (0.13–5.88)			0	
CRF		0.32 (0.06–1.68)			0	
Diabetes		1.29 (0.87–1.92)			2.47 (0.98–6.23)	
Malignancy		1.74 (0.96–3.18)			0.39 (0.04–3.65)	
UGIB		1.30 (0.06–29.27)			0	
Infection		1.03 (0.82–1.30)			1.53 (1.01–2.33)	1.67 (1.16–2.40)
IBD		1.08 (0.36–3.24)			0.75 (0.21–2.66)	
Psoriasis		0.70 (0.18–2.69)			0.77 (0.07–8.51)	
Extra-Articular RA		1.64 (0.96–2.80)				
**Non-Mental Health Care Utilization in Past 2 Years**						
Physician Visit		2.25 (1.57–3.21)	2.30 (1.64–3.24)		1.42 (0.83–2.43)	1.46 (0.89–2.37)
Rheumatology Visit		1.03 (0.70–1.52)			0.54 (0.27–1.11)	
Hospitalization		1.43 (0.97–2.13)			1.35 (0.68–2.68)	
ED Visit		1.41 (1.11–1.79)	1.50 (1.20–1.88)		2.14 (1.44–3.20)	2.49 (1.74–3.56)

Model ^a^: adjusted for income, ethnicity, previous physician visit, previous ED visit, a history of psoriasis or a history of chronic obstructive pulmonary disease.

Model ^b^: adjusted for income, ethnicity, previous physician visit, previous ED visit and a history of infection.

ARF, acute renal failure; AS, ankylosing spondylitis; CAD, coronary artery disease; COPD, chronic obstructive pulmonary disease; CRF, chronic renal failure; CVD, cardiovascular disease; ED, emergency department; IBD, inflammatory bowel disease; MI, myocardial infarction, OA, osteoarthritis; OP, osteoporosis; RA, rheumatoid arthritis; UGIB, upper gastrointestinal bleed

## Discussion

To our knowledge, this is the first population-based study to examine rates and risk for an initial DSH attempt among individuals with RA, and the largest of its kind in an AS population. This study was novel because of our ability to evaluate methods of self-harm and disposition following DSH in these two prototypical inflammatory conditions. Both RA and AS showed a small, but increased incidence of DSH compared to individuals without RA or AS. Our key finding was a 59% increase in the risk of DSH associated with AS. This is important because AS is a rare condition but was independently associated with a health outcome with significant health and societal cost [16, 20]. Our findings also suggest that there may be a differential impact, by arthritis diagnosis, on the risk of DSH.

We found the absolute rate of DSH to be small in the RA, AS and comparator cohorts. In their paper examining depression and suicide, Wu and colleagues showed a crude rate of non-fatal suicide (defined by inpatient and outpatient diagnostic codes) in AS to be 1.48/1,000 PY [10]. A plausible reason for our lower rate of 0.68/1,000 PY is that we only included the first DSH attempt and DSH had to be severe, requiring presentation to the ED, not outpatient encounters. We also excluded subjects with any prior mental illness or self-harm attempt. Combined, these exclusions may account for our observed low event rates. DSH obtained from administrative data is also likely an under estimate of the true problem, as not all patients seek attention, or they may not disclose that an injury was self-inflicted [17].

The possible mechanisms explaining this association are unknown. Studies of other physical illnesses and suicidal ideation point to depression as a major precursor of DSH [18, 19]. The most common comorbidity in RA is in fact depression but we did not observe a greater adjusted risk for DSH in our RA sample [2, 35]. A recent study demonstrated that 31% of AS patients report moderate-to-severe depressive symptoms [36]. Others have shown that depressive symptoms are severe in males with AS and may intensify following AS diagnosis [10, 37]. Our study did not have data on depression or disease severity; however, our AS subjects were primarily male and younger compared to other cohorts. This may represent a particularly high risk group for severe depression [12].

Inflammatory cytokines are purported to play a role in the association between depression and self-harm attempts. Inflammatory arthritis has increased levels of interleukins (IL; IL-1, IL-2, and IL-6) and tumor necrosis factor alpha in the systemic circulation and joints [38, 39]. These same cytokines are present in high levels among individuals without arthritis who have attempted suicide [40]. Therefore, aberrant cytokine levels in the central nervous system may cause physiologic and biochemical changes that contribute to self-harm behaviour [3, 40]. Of interest, a review by Ganança et al. demonstrated that elevated IL-6 in cerebrospinal fluid and serum was associated with suicidal ideation [3]. Studies of psoriasis and psoriatic arthritis, conditions that are closely linked with AS, have suggested that elevated IL-17 may increase the risk of severe depression and suicide intent [38, 41]. AS typically develops at a younger age than RA, and AS symptoms may precede formal diagnosis by years. In fact, the mean age (SD) at time of AS diagnosis in our cohort was 46.4 ± 16.6 years, which suggests a significant lag in diagnosis by decades. In this setting, AS patients may have a long period of susceptibility to develop the psychological effects of inflammatory cytokines. Whether immune mechanisms are mediators of DSH behavior cannot be ascertained by our data, and is likely a complex phenomenon with biological, psychological and social interaction that will require further hypothesis testing.

Other notable findings are worth mentioning. One was our ability to characterize the method of DSH. We found a higher proportion of self-harm attempts in AS subjects by self-cutting. Similar to the general population, this method is a recognized risk factor for repeat DSH and subsequent suicide [17, 42]. Poisoning was the method accountable for 81% of self-harm attempts in RA patients and slightly lower in AS (64%). We were unable to determine if these were intentional or accidental overdoses of prescription medications. Since the mainstay of treatment in both RA and AS is medical therapy, the types and access to prescription medications may be a target for safeguards in high risk individuals. Second, more than two-thirds of patients were discharged from the ED following their self-harm attempt. We cannot ascertain the quality of the care received in the ED, or if any psychiatric care was implemented or prescribed for the outpatient setting [16]. Further investigation into whether an ED visit can prevent subsequent DSH, or if patients with RA or AS are prone to repeated DSH attempts, is another important area to study.

Our study has several strengths. The large sample size allowed us to generate confidence around our estimates. We had long follow-up to ascertain rare events over time. We used established algorithms to define our cohorts and outcome to reduce the risk of misclassification. The population-based setting reflects clinical practice and enhances the generalizability of our findings. The limitations of our study relate to the level of detail in administrative health data. We could not include variables relevant to DSH such as depressive symptoms, suicidal intent, impulsivity, or substance abuse with alcohol, cigarettes or illicit substances [16]. We were unable to adjust for unmeasured confounders such as RA or AS disease severity, social isolation or disability. We did not have medication records (including disease modifying antirheumatic drugs, or mental health medications) as only a small proportion of patients were over the age of 66 for whom prescription data are universally available. We did not explore time-varying factors such as mental health diagnoses or health care visits in the interval after RA/AS diagnosis that may have influenced a DSH attempt. However, medication use that may influence self-harm behaviours (e.g. opioids, prednisone) or mental health illnesses after RA or AS diagnosis (e.g. personality disorders) would be mediators along the causal pathway and would not be expected to confound the association between arthritis diagnosis and self-harm.

We conclude that a diagnosis of AS, carries a small, but significantly increased risk for DSH. No such risk was observed following a diagnosis of RA in our data, but this should be verified in other cohorts. With this knowledge, future efforts should focus on identifying characteristics of AS subjects that most strongly predict risk and determine the vulnerability of repeated DSH attempts, which are frequently lethal [42]. Whether targeted treatments for inflammatory arthritis change rates of DSH over time is also needed. Finally, rheumatologists and primary care physicians closely follow patients with RA and AS. These medical settings can provide potential opportunities for screening and triage of mental illness, including self-harm behavior. To facilitate this, accurate methods of screening must be available and feasible in order to determine if the prevalence of DSH in routine clinical practice is significant enough to warrant the design of specific risk-reduction strategies for individuals with inflammatory arthritis.

## Supporting information

S1 Dataset Dataset creation plan. (PDF)
