# Supplementary material for: The risk of deliberate self-harm following a diagnosis of rheumatoid arthritis or ankylosing spondylitis: A population-based cohort study
Source: PLoS One. 2020 Feb 21;15(2):e0229273. doi: 10.1371/journal.pone.0229273 (PMC7034875; doi:10.1371/journal.pone.0229273)
Supplement: S1 Dataset — (PDF) [file pone.0229273.s001.pdf]

Project Initiation
This Section must be Completed Prior to Project Dataset(s) Creation

**Project Title:**	Mental Health Service Use and Serious Mental Health Outcomes for Individuals with Rheumatoid Arthritis , Ankylosing Spondylitis or Psoriatic Arthritis
**Project TRIM number:**	
**Research Program:**	CDP
**Site:**	ICES Central
**Project Objectives:**	*Insert Project Objectives as listed in the approved ICES Project PIA*<br>**1)** To evaluate the frequency and types of mental health service use **following** RA and AS diagnosis (separately) compared to the general population;<br>**2)** To assess the risk of serious mental health outcomes among RA and AS<br>**3)** To determine what clinical and demographic variables are associated with serious mental health outcomes following RA or AS diagnosis
**ICES Project PIA Initial Approval Date:**	*The ICES Employee or agent who is responsible for creating the Project Dataset(s) is responsible for ensuring there is an approved ICES Project PIA and verifying the date of approval prior to creating the Project Dataset(s)*<br>yyyy-mon-dd
**Principal Investigator (PI):**	Bindee Kuriya
**Check the applicable box if the PI is an ICES Student/Trainee**	☐ ICES Student ☐ ICES Fellow ☐ ICES Post-Doctoral Trainee ☐ Visiting Scholar
**Responsible ICES Scientist:**	*Name the Responsible ICES Scientist if the PI is not a Full Status ICES Scientist*<br>Simone Vigod
**Project Team Member(s) Responsible for Project Dataset Creation and/or Statistical Analysis and date joined (list all):**	*All person(s) (ICES Analyst, Appointed Analyst, Analytic Epidemiologist, PI, and/or Student) responsible for creating the Project Dataset(s) and/or statistical analysis on the Research Analytics Environment (RAE) <u>and the date they joined the project</u> must be recorded*<br>yyyy-mon-dd
**Other ICES Project Team Members and date joined (list all):**	*All other Research Project Team Members (e.g., Research Administrative Assistants, Research Assistants, Project Managers, Epidemiologists) <u>and the date they joined the project</u> must be recorded*<br>yyyy-mon-dd
**Confirmation that DCP is consistent with Project Objectives:**	*The following individuals must confirm that the ICES Data provided for in this DCP is relevant (e.g., with respect to cohort, timeframe, and variables) and required to achieve the Project Objectives stated in the ICES Project PIA <u>prior to initial Project Dataset creation</u>: 1) PI; 2) Responsible ICES Scientist if the PI is not a Full Status ICES Scientist, or a second ICES Scientist or the Scientific Program Lead if the PI is creating both the DCP and the Project Dataset[s]; 3) ICES Research Practice Staff creating the DCP; and 4) ICES Analytic Staff (ICES Employee or agent responsible for creating the Project Dataset[s]). This may be delegated either verbally or via e-mail.*<br>***Principal Investigator*** ☐ yyyy-mon-dd<br>***Responsible ICES Scientist or Second ICES Scientist/Lead*** ☐ yyyy-mon-dd<br>***ICES Research Practice Staff Creating the DCP*** ☐ yyyy-mon-dd<br>***ICES Analytic Staff*** ☐ yyyy-mon-dd
**Designated ICES Research Practice Staff accountable for Project Documentation:**	*The person named (ICES staff) is accountable for ensuring that the approved ICES Project PIA, ICES Project PIA Amendments, and DCP are saved on the T Drive, ensuring ICES Project PIA Amendments are submitted as required, ensuring DCP Amendments are documented, and sharing the final DCP with the PI/Responsible ICES Scientist at project completion*

Project Initiation
This Section must be Completed Prior to Project Dataset(s) Creation

**DCP Creation Date and Author:**	*Date DCP was finalized prior to Project Dataset(s) creation*	*Name of person who created the DCP*
	Date	***Name***
	Updated Jan.8, 2018	Bindee Kuriya

ICES Data<br>This Section must be Completed Prior to Project Dataset(s) Creation	
The ICES Employee or agent who is responsible for creating the Project Dataset(s) must ensure that this list includes only data listed in the ICES Project PIA<br>*Changes to this list after initial ICES Project PIA approval require an ICES Project PIA Amendment*	*Mandatory for all datasets that are available by individual year*
General Use Datasets – Health Services	***Years (where applicable)***
CIHI DAD	1st April 2002 - 31st March 2016
OHIP	1st April 2002 - 31st March 2016
General Use Datasets – Care Providers	
IPDB	1st April 2002 - 31st March 2016
See list	
General Use Datasets – Population	
RPDB	1st April 2002- 31st March 2016
POP	1st April 2002 - 31st March 2016
General Use Datasets – Coding/Geography	
LHIN	1st April 2002 - 31st March 2016
See list	
General Use Datasets - Facilities	
See list	
General Use Datasets - Other	
ORAD	1st April 2002- 31st March 2016
See list	
Controlled Use Datasets	
ETHNIC	1st April 2002- 31st March 2016
See list	
Other Datasets	
**NACRS (2002 on),** ODB, ORGD, OCR, ODD, ORAD, Statistics Canada Census, OMHRS	1st April 2002 - 31st March 2016

Project Amendments and Reconciliation

**ICES Project PIA Amendment History (add additional rows as needed):**	*Privacy approval date*	*Person who submitted amendment*	*Note that any changes to the list of ICES Data or Project Objectives require an ICES Project PIA Amendment*
	Date	***Name***	***Amendment***
	yyyy-mon-dd		
**DCP Amendment History (add additional rows as needed):**	*Date DCP amended*	*Person who made the DCP amendment*	*Note that any DCP amendments involving changes to the list of ICES Data or Project Objectives require an ICES Project PIA Amendment*
	Date	***Name***	***Amendment***
	yyyy-mon-dd		
**Date Programs/DCP reconciled**	*The person(s) creating the dataset and/or analyzing the data are responsible for ensuring that the final DCP reflects the final program(s) when the project is completed*		
	yyyy-mon-dd		

Project Cohort

Study Design

☐ Cohort study ☒ Matched cohort study ☐ Case-control study

☐ Cross-sectional study ☐ Other (specify):

Index Event / Inclusion Criteria

Exposure study group 1: **Incident RA patients defined in ORAD database since 1996:**

- At least 1 CIHI-DAD claim with ICD9 dx 714.x
  OR
- 3 OHIP claims dx 714 with at least 1 OHIP dx 714.x by rheumatologist within 2 year period [IPDB MAINSPECIALITY = RHEUMATOLOGY]
-

Unexposed study group 1: **no RA diagnosis in prior 2 years according to ORAD definition,** 4:1 matched on age (+/- 2 yr), sex and calendar year with controls randomly assigned entry dates matching the distribution found in those with RA

____

Exposure study group 2: **Incident AS patients defined as:**

- At least 2 OHIP claims with OHIP dx 720 within 2 years, with at least 1 claim by a rheumatologist [IPDB MAINSPECIALITY = RHEUMATOLOGY]
  or
- At least 1 CIHI-DAD claim with ICD9 dx 720.x or ICD10 dx M45x (any diagnosis type)

Unexposed study group 2: **no AS diagnosis in prior 2 years,** 4:1 matched on age (+/- 2 yr), sex and calendar year with controls randomly assigned entry dates matching the distribution found in those with AS

Estimated Size of Cohort (if known)

ORAD (approximately 98,000 RA patients, ~5000 incident cases per year
AS cohort (approximately 40,000)

Exclusions (in order)

Step	Description
1	**Study group 1:** Exclude if other systemic connective tissue disorders (SLE, Scleroderma, Sjogren's, myositis) or inflammatory arthritis (psoriatic arthritis, AS) in 2 years before index date

Project Cohort		
		ICD9: 710x, 696x, 720x<br>ICD10: M32-M36x, L40x, M45x<br><br>**Study group 2:** Exclude if other systemic autoimmune rheumatic disease (SLE, Scleroderma, Sjogren's, myositis) or inflammatory arthritis (psoriatic arthritis, rheumatoid arthritis) in 2 years before index date<br>ICD9: 710x, 696x, 714x<br>ICD10: M32-M36x, L40x, M05x
	2	**Excldue mental health care use or serious mental health outcomes in 2 years** prior to baseline (exclude ull all outpatient,hospitalization or ER visits (LOCATION = O/H/L) to a FP/GP [OHIP spec = 00] and [OHIP spec=19] with a MHA diagnostic code (OHIP 290-315)
	3	**Non-ON residents or Invalid IKN**
	4	**Missing age or sex on entry**

Project Time Frame Definitions

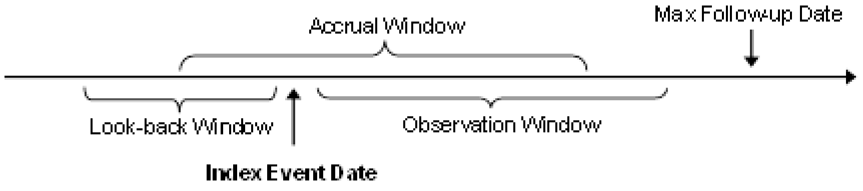

**Accrual Start/End Dates**	1st April 2002- 31st March 2014 (with minimum 2 years of follow-up)
**Max Follow-up Date**	31st March 2016
**When does observation window terminate?**	Censor if any of the following occur during follow up to the end of the study period (31st March, 2013):<br>• Death<br>• Outcome event (self-harm ER visit, or hospitalization for self-harm)<br>• Loss of OHIP coverage (Jin did not use this)<br>• Control group diagnosis of RA or AS (This person will be censored in the comparison arm but will then be eligible to enter the RA or AS cohort)<br>• March 31, 2016
**Lookback Window(s)**	Comorbidities: 24 months preceding entry (except for ICES-derived chronic disease cohorts)

Variable Definitions (add additional rows as needed)	
**Main Exposure or Risk Factor**	Rheumatoid arthritis (study 1)<br>Ankylosing spondylitis (study 2)
**Primary Outcome Definition**	**Outpatient Mental Health Service Use (Appendix 1)**<br>• Pull all outpatient visits (LOCATION = O/H/L) to a FP/GP [OHIP spec = 00] and [OHIP spec=19] with a MHA diagnostic code (OHIP 290-315)<br>• Health care use according to diagnostic mental health categories
**Secondary Outcome Definition(s)**	Serious mental health consequences:<br>1) **Hospitalization for mental health disorder** and according to diagnostic mental health categories **(APPENDIX 2)**<br>2) **ED visit for mental health disorder** (no admission) and according to diagnostic mental health categories **(APPENDIX 3**)<br>3) **ER visit for self-harm** (self-harm definition 1 or 2) **(APPENDIX 4)**<br>-method of self-harm and<br>-disposition following attempt
**Baseline Characteristics**	**1.** For all members (RA group, AS group and controls)<br>**2.** Age at entry (from RPDB)<br>**3.** Sex (from RPDB)<br>**4.** Ethnicity: South Asian, Chinese or Caucasian using surname file (Shah 2012)<br>**5.** Income quintile based on median household income level of the individual's neighborhood of residence using the Census year closest to entry.<br>a. April 1 1993 - March 31 1999: Use 1996 EA<br>b. April 1 1999 - March 31 2004: Use 2001 DA<br>c. April 1 2004 - March 31 2009: Use 2006 DA<br>**6.** LHIN<br>**7.** Rurality index of Ontario<br>**8.** Extra-articular features for RA only (Appendix 1)<br>**9.** Comorbidity index (Charlson): missing, 0, 1, 2, 3+<br><br><u>**Individual comorbidities**</u> **(Appendix 5. Use ICES-derived Cohorts unless otherwise indicated)**<br>**10.** Osteoarthritis<br>**11.** Osteoporosis<br>**12.** COPD/Asthma (ICES cohorts) use both the sensitivity and specific definitions<br>**13.** CAD, acute MI<br>**14.** Cerebrovascular disease<br>**15.** Hypertension (ICES cohort)<br>**16.** Acute renal failure<br>**17.** CKD/Chronic Dialysis<br>**18.** Diabetes (ODD)<br>**19.** Dementia (use current up to date)<br>**20.** Ontario cancer registry (separate skin and other cancers)<br>**21.** Upper GI bleed<br>**22.** Infections: Hospitalization with a "most responsible" (primary) diagnosis of infection,<br>**23.** Inflammatory bowel disease<br>**24.** Hypothyroidism

Variable Definitions (add additional rows as needed)	
	**Health Services Utilization**<br>**25.** Resource utilization band (qunitiles of health care use)<br>**26.** No. of unique drug products<br>**27.** No. of emergency department visits<br>**28.** No. of days in hospital<br>**29.** Access to rheumatology care (consider time from diagnosis to first rheumatology visit [if different than diagnostic encounter]; RHEUMATOLOGIST: IPDB MAINSPECIALTY="RHEUMATOLOGY")
**Other Variables**	**TIME-DEPENDENT VARIABLES**<br><br>**ALL**<br>**a)** Non-mental health care use (GP visits, hospitalizations)<br>**b)** Number of rheumatology follow up visits<br>**c)** Number of individual comorbidities<br><br>**RA ONLY**<br>**d)** Extra-articular features (for RA patients only)

Analysis Plan and Dummy Tables (expand/modify as needed)

**Descriptive Tables (insert or append dummy tables), e.g.:**	
**Table 1. Baseline characteristics (Table 1)**	
**Table 2. Outpatient mental health care use (table 2 and figures)**	
**Table 3and 4 . MH hospitalizations and ED visits (Tables 3,4)**	
**Table 5. Self-harm**	
**Statistical Model(s)**	
**Type of model**	Primary outcome: cumulative incidence rates (annually for years after diagnosis) of outpatient mental health use in RA and AS separately<br><br>Secondary outcomes: crude and adjusted Cox proportional hazards models, adjusted for baseline and time-varying covariates to assess risk factors for (1) hospitalizations; (2) ED visits only; (2) self harm RA and AS separately
**Primary independent variable**	RA<br>AS
**Dependent variable**	Model 1: hospitalization<br>Model 2: ER visit only<br>Model 3: ER visit for self-harm
**Covariates**	Baseline variables and time varying covariates as listed above

Quality Assurance Activities

**RAE Directory of SAS Programs**	

Quality Assurance Activities

**RAE Directory of Final Dataset(s)**	*The final analytic dataset for each cohort includes all the data required to create the baseline tables and run all the models. It should include all covariates for all models such as patient risk factors, hospital characteristics, physician characteristics, exposure measures (continuous, categorical) and outcomes. It should include covariates that were considered but didn't make the final cut. This would permit an analyst to easily re-run the models in the future.*

RAE README file available: ☐Yes ☐No

Date results of quality assurance tools for final dataset shared with project team (where applicable):

	**%assign**	yyyy-mon-dd
	**%evolution**	yyyy-mon-dd
	**%dinexplore**	yyyy-mon-dd
	**%track / %exclude**	yyyy-mon-dd
	**%codebook**	yyyy-mon-dd

Additional comments:
